# Antibiotic Prescribing Trends in Dentistry during Ten Years’ Period—Croatian National Study

**DOI:** 10.3390/antibiotics13090873

**Published:** 2024-09-12

**Authors:** Ivana Šutej, Krešimir Bašić, Sanja Šegović, Kristina Peroš

**Affiliations:** 1 Department of Pharmacology, School of Dental Medicine, University of Zagreb, 10000 Zagreb, Croatia; isutej@sfzg.hr (I.Š.); basic@sfzg.hr (K.B.); 2 Department of Endodontics and Restorative Dentistry, School of Dental Medicine, University of Zagreb, 10000 Zagreb, Croatia; segovic@sfzg.hr

**Keywords:** antibiotics, antimicrobial resistance, odontogenic infection, amoxicillin, prescriptions

## Abstract

Prescribing antibiotics is a regular part of daily dental practice. Antibiotics have a significant but a limited role in general dental practice due to the threat of emergence of antimicrobial resistance (AMR). As such, the aim of this study was to assess prescribing trends in dental antibiotics use from 2014–2023 in Croatia. Data on antibiotic prescribing practices for this study were provided by the Croatian Health Insurance Fund. The analysis included the number of prescriptions, packages, cost, and the World Health Organization’s defined daily dose per 1000 inhabitants (DID) per day as an objective utilization for comparison. Over the 10-year period, dentists in Croatia prescribed an annual average of 357,875 antibiotic prescriptions, representing an annual average of 78.7% of all dental prescriptions. The most commonly prescribed antibiotic was the combination of amoxicillin and the beta-lactamase inhibitor clavulanic acid, which made up 58.54% of antibiotics and 46.1% of all dental prescriptions. This was followed by amoxicillin (12.61%), clindamycin (12.58%), and metronidazole (9.96%). The trend showed two discontinuations, the first for the pandemic years, and the second caused by disruption in amoxicillin production. The rise in the use of broad-spectrum antibiotics needs to be addressed and regulated to ensure patients and dentists understand that antibiotics are not a substitute for dental treatment. Dentists should always begin treatment with narrow-spectrum antibiotics regardless of possible exceptional circumstances.

## 1. Introduction

Prescribing medications is a regular part of dentists’ daily practice. Among prescribed medications, antibiotics are the most prescribed. Most common infections that need antibiotic therapy in dentistry have odontogenic and periodontal origins and are regulated by the guidelines brought about and updated regularly by their societies [1,2]. In these cases, systemic antimicrobial therapy in dentistry serves solely as adjunct to mechanical treatment. The first-choice antibiotic for endodontic or odontogenic infections is beta-lactam antibiotic amoxicillin. If ineffective after 48–72 h, metronidazole can be added, or treatment can be switched to amoxicillin/clavulanic acid. For patients allergic to penicillin, clindamycin is recommended [2]. In periodontitis, systemic antimicrobials are used alongside scaling and root planing, particularly in cases of persistent or recurrent disease, early-onset disease, or uncontrolled diabetes. The preferred antibiotic therapy includes amoxicillin with metronidazole or, much less commonly, metronidazole with azithromycin [1]. Besides treatment of odontogenic infection, antibiotics in dentistry can be used prophylactically for patients at risk, as well as in implantology [3,4]. When not used as indicated, the chances for antimicrobial resistance development increases. Antimicrobial resistance represents a huge global problem, while the dental community bears the burden of responsibility, with an average share of 10% of national antibiotic outpatient prescriptions [5]. However, as far as inappropriate antibiotic use is concerned, dentists often describe it as a “gray area”: decision making under uncertainty, ethical challenges associated with the clinical judgment varying from patient to patient, depending on their vulnerability and risk population they are in, and clinical decisions that depend on the moral judgment of individual prescribers varying in their antibiotic-prescribing approach [6,7]. Therefore, to minimize uncertainty and misconduct, Sandulescu proposed the 5Ds, following key questions that should be asked before each antibiotic prescription [7]: What dental treatment should I perform? Is an antibiotic needed for this dental condition (clinical indication)? Is this the narrowest possible antibiotic drug? Is this the correct dose? Is this the shortest duration?

As for the dental antibiotic utilization in Croatia, there are a couple of weak spots to be addressed. First is the dominance of co-amoxiclav as the most frequently prescribed antibiotic [5]. Since current guidelines on the use of antibiotics in dental medicine recommend starting antibiotic therapy with a narrower-spectrum antibiotic, the authors made significant efforts to change this trend to align with reported antibiotic prescribing practices in other EU countries. The effort included lectures and seminars for pre-graduate dental students, new lectures and problem-based learning practicals for postgraduate specialist studies, lectures in continuing education courses for dentists with scored points approved by the Croatian Dental Chamber, and two public radio shows. Following mentioned interventions, some improvements in antibiotic prescribing trends were noticed but not published. In the winter of 2022–2023, a shortage in the production of the main antibiotics emerged due to changing infection patterns resulting in an increased demand [8]. The shortage was for a short time period and was announced in advance by the major Croatian drug producer, Pliva/Teva company, but the consequences have not been investigated.

The aim of this study was to assess the antibiotic prescribing patterns of Croatian dental practitioners from 2014 to 2023 and to examine how production shortages influenced their prescribing decisions.

## 2. Results

### 2.1. General (Overall) Data

During the analyzed period, dentists issued an average of 454,789 prescriptions annually, showing a 6.1% increase from 2014 to 2023 (Table 1). The average number of licensed dental practices in Croatia during the investigated period was 2556, with the number of dentists increasing by 5% during this period. The number of patients that used dental services was, on average, 1,633,710 per year with some minor annual fluctuations during the pandemic years (2020 and 2021). In 2014, Croatia’s population was 4,238,389, which decreased by 9.1% over the next decade [9].

### 2.2. Antibiotic Utilization

Analyzing dental prescriptions by ATC classification, antibacterials (J01) were the most frequently prescribed drugs, constituting an average of 70.9% of all prescriptions. When including metronidazole (P01A), a key choice for anaerobic infections in dentistry, the combined total average represented 78.7% of all annual prescriptions. The most commonly prescribed antibiotic was the combination of amoxicillin and the beta-lactamase inhibitor clavulanic acid, which made up 58.54% of antibiotics and 46.1% of all dental prescriptions. This was followed by amoxicillin (12.61%), clindamycin (12.58%), and metronidazole (9.96%) (Table 2).

When expressing these data in antibiotic prescriptions/1000 population, it would be 56 for co-amoxiclav, 12 for clindamycin, 8.7 for amoxicillin, and 7.4 for metronidazole, while in total, dentists are responsible for 89.2 antibiotic prescriptions per 1000 population in Croatia. Results expressed in DID are presented in Table 3.

**Figure 1:**
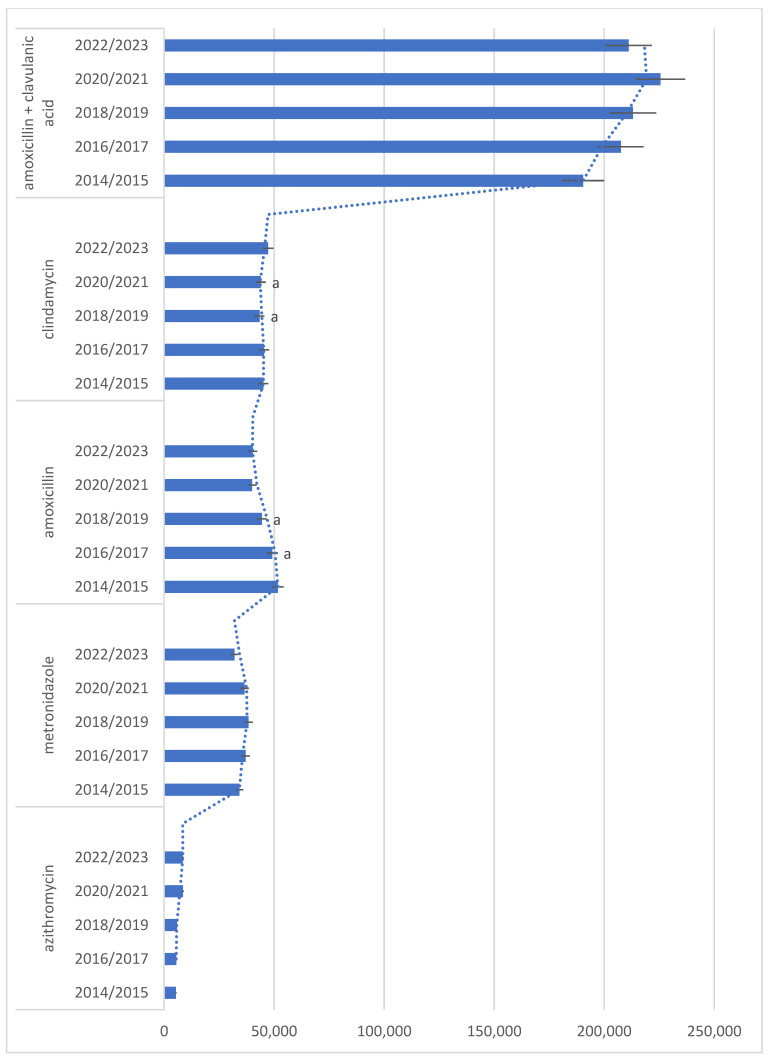
Descriptive statistics of the most commonly prescribed antibiotics in time intervals (2014/15, 16/17, 18/19, 20/21, 22/23). Same letters (a) indicate statistically significant values for comparisons among year intervals—for clindamycin (2018/2019 compared to 2020/2021, *p* = 0.045) and for amoxicillin (2016/2017 compared to 2018/2019, *p* = 0.017). Bars denote mean value of number of prescriptions for each time interval. Error bars denote ± 1 SD. Dotted line denotes trend line—two period moving average.

**Figure 2:**
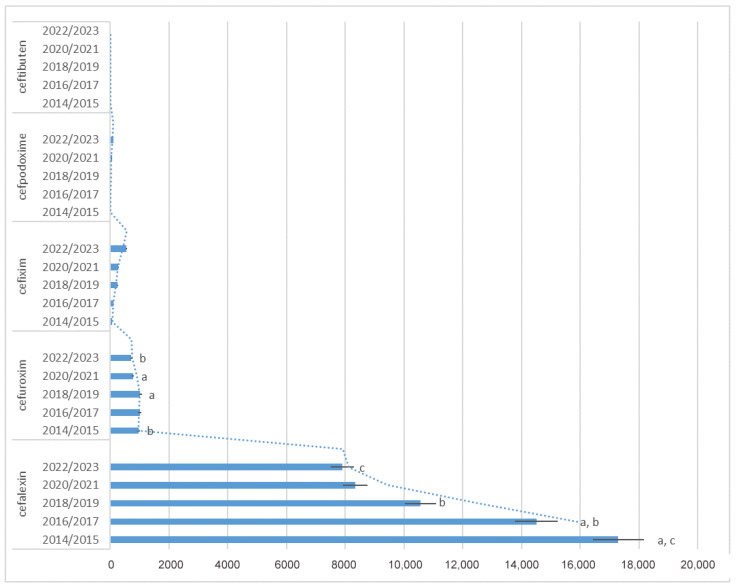
Descriptive statistics of prescribed cephalosporins in time intervals (2014/15, 16/17, 18/19, 20/21, 22/23). Same letters (a, b, c) indicate statistically significant values for comparisons among year intervals—for cefalexin (2014/2015 compared to 2016/2017, *p* = 0.017; 2016/2017 compared to 2018/2019, *p* = 0.033; 2014/2015 compared to 2022/2023, *p* = 0.027) and for cefuroxim (2018/2019 compared to 2020/2021, *p* = 0.009; 2014/2015 compared to 2022/2023, *p* = 0.014). Ceftibuten was discontinued in 2017. Bars denote mean value of number of prescriptions for each time interval. Error bars denote ± 1 SD. Dotted line denotes trend line—two period moving average.

**Figure 3:**
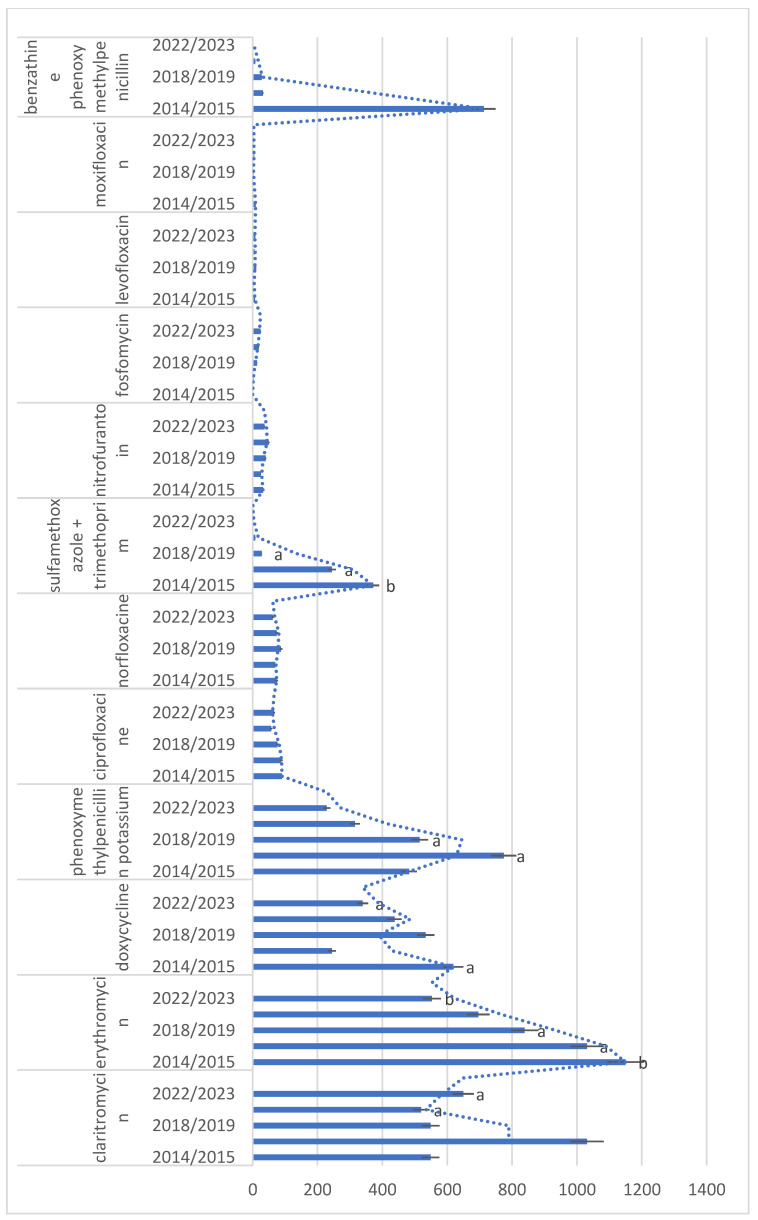
Descriptive statistics of antibiotics with less than 1400 prescriptions per year, in time intervals (2014/15, 16/17, 18/19, 20/21, 22/23). Same letters (a, b) indicate statistically significant values for comparisons among year intervals—for the interval 2016/2017 when compared to 2018/2019 (levofloxacin *p* = 0.042; sulfamethoxazole + trimethoprim *p* = 0.048; phenoxymethylpenicillin potassium *p* = 0.033; erythromycin *p* = 0.002), for the interval 2014/2015 when compared to 2022/20223 (sulfamethoxazole + trimethoprim *p* = 0.001; doxycycline *p* = 0.047 and erythromycin *p* = 0.021) and for the interval 2020/2021 when compared to 2022/2023 (claritromycin *p* = 0.039). Bars denote mean value of number of prescriptions for each time interval. Error bars denote ± 1 SD. Dotted line denotes trend line—two period moving average.

Results on descriptive statistics of the most commonly prescribed antibiotics in time intervals (2014/15, 16/17, 18/19, 20/21, 22/23) are presented in Figure 1, Figure 2 and Figure 3, with results on statistical analysis of comparisons among year intervals. Analyses showed statistically significant difference in amoxicillin, clindamycin, and cephalosporins antibiotics frequency of prescribing in comparison years, but also in other antibiotics, as shown in Figure 3.

### 2.3. Dentists Share in National Antibiotic Consumption

Dental antibiotic prescriptions accounted for 3.3% of the total national antibiotic consumption and 9.6% of outpatient national consumption in 2023. Their contributions to outpatient antibiotic use included the highest proportions for co-amoxiclav (27%), clindamycin (21%), metronidazole (19.2%), and amoxicillin (16%) [10]. On average, each dental practice issued 133 antibiotic prescriptions annually with an average number of 0.2 antibiotic prescriptions per insured patient who received some kind of dental service. The Croatian Health Statistics Yearbook and annual reports from the CIPH indicate that the number of odontogenic infections (classified under K04 in the International Classification of Diseases-10th edition) and periodontal infections (classified under K05,2-K05,4) decreased by 17% and 11.5%, respectively, over the observed time period.

### 2.4. Changes in Utilization Trend

The total number of dentists’ antibiotics prescriptions increased by 24,220 until 2021 (6.9%), followed by the decrease in total antibiotic prescription of 13,541 in 2022 (3.5%), and an additional decrease of 13,155 in 2023 (3.7%). A trend discontinuum was also observed in the first pandemic year when the total number of antibiotics prescribed by dentists increased by 7744 (2.4%). The major difference in pandemic years was an increase in azithromycin prescribing, which was specific only for that period [11]. In 2023, a sharp fall in amoxicillin prescriptions of 28.6% was observed, representing its lowest number for the investigated period.

## 3. Discussion

Based on data from 2014–2023, the major finding of this study was that the proportion of antibiotic use among dentists in Croatia is high and inappropriate. More than half of the prescribed antibiotics (63%) were wide spectrum co-amoxiclav, although all available guidelines in dentistry recommend amoxicillin as the first-choice antibiotic. The same result was confirmed with two questionnaire studies and two prospective studies conducted previously in Croatia [12,13,14,15]. Co-amoxiclav is the third-choice antibiotic in dentistry according to relevant guidelines [1,2] and is therefore an inappropriate first prescribing choice, mostly because of its broad-spectrum activity and potential for resistance development.

First-choice antibiotics by dentists differ between countries. Co-amoxiclav is the first choice in Spain [16], Turkey [17], and Romania [18], with a similar proportion among other antibiotics as in our study. Spain has also showed differences among studies, with the newest study showing an improved ratio between amoxicillin and co-amoxiclav [19]. In Serbia, the first-choice antibiotic depended on the dentists age, with older dentists prescribing co-amoxiclav and younger prescribing amoxicillin [20]. In Norway and Sweden [21], the first choice is penicillin V, although they have noticed a surge in co-amoxiclav prescriptions lately [22]. Most European countries [23,24,25], as well as Australia, Canada, and the US [26,27,28], have amoxicillin as the first choice, which is in accordance with available guidelines [1,2]. The excessive and inappropriate utilization of antibiotics among Croatian dentists may be associated with the training and experiences of doctors, as well as the knowledge and attitude of patients towards antibiotics. A latest study on reasons for inappropriate prescribing revealed that using broad-spectrum antibiotics when not indicated and prescribing without proper examination was directly associated with inadequate knowledge of dental ethics. Given explanations by dentists for such a behavior were perceived expectations of patients, use as a precautionary measure to prevent unlikely complications in healthy patients after non-surgical procedures, and choosing precautionary broad-spectrum over narrow-spectrum antibiotics against recommended protocols [6] just to be sure that they covered all possible antibacterial spectra. The only way to correct such behavior is education and antibiotic stewardship programs, which will increase security in making the right choice of antibiotic; this can be supported by educating patients as well.

Comparisons between year intervals in our study pointed to three main periods of significant changes in prescribing patterns: pre-COVID (2014–2018), during COVID (2019–2021), and post-COVID (2022–2023). Each of these periods had specificities for each antibiotic or antibiotic group, with prescribing differences related to changed circumstances and emerging consequences of solving new challenges. Some low consumption antibiotics during whole period (2014–2023) were even more decreased in number of prescriptions (e.g., erythromycin, doxycycline, sulfamethoxazole with trimethoprim, phenoxymethylpenicillin potassium) while others increased (e.g., clarithromycin, co-amoxiclav).

Our study found a sharp fall in amoxicillin prescriptions, 28.6%, observed in 2023, representing its lowest number for the 2014–2023-time span, followed by an unwanted increase of 6% in co-amoxiclav prescriptions. These results indicated that dentists in Croatia responded to these exceptional circumstances by switching prescribing choice to co-amoxiclav, instead of similar spectrum available antibiotics such as cephalosporins, adding to the already overburdened co-amoxiclav utilization. These findings are in concordance with several studies reporting decrease in amoxicillin prescriptions in 2023 in various healthcare departments [29,30,31]. Health authorities and public health agencies across Europe and the US reported shortages of amoxicillin during the winter of 2022/2023, despite antibiotic shortages not previously being considered a critical issue in the public health of Western countries. Our results indicate that Croatia was not exempt either. The main reasons for any drug shortage usually lie in reduced production, disturbed distribution, or both. Cohen et al. proposed the cause and effect for amoxicillin shortage in this period as the need for amoxicillin had been diminished during the recent coronavirus pandemic, which led to a decrease in production lines. Stocks of amoxicillin were insufficient to meet the needs, so physicians turned to prescribing other available antibiotics. The recovery of production capacity to levels before pandemic was too slow, compounding the shortage as well as the risk of widespread antibiotic shortages [29]. Poole et al. quantified antibiotic prescribing for ambulatory pediatric acute respiratory illness in periods before and after amoxicillin shortage and found that amoxicillin prescriptions decreased, overall prescribing patterns changed, and azithromycin index was shown without change [30]. Our results are mostly in concordance with their findings for amoxicillin. The need for action is recognized, so the European Commission, the Heads of Medicines Agencies (HMA), and the European Medicines Agency (EMA) issued recommendations for steps and actions to avoid shortages of key antibiotics used to treat respiratory infections for European patients in the forthcoming winter season [32].

The compliance with the main principles of antimicrobial stewardship in dentistry should be consistent even through suddenly changing circumstances such as temporary antibiotic shortage in production or distribution. We cannot emphasize enough that antibiotic therapy is not a substitute for dental treatment but rather an adjunct to operative intervention or other treatment modalities of dental infection, so an antibiotic should be prescribed only if there is an evident clinical benefit. Despite this, prompt usage of effective antibiotics and operative management in treating severe, spreading dental infection remains an imperative. The choice of the most narrow-spectrum antimicrobial that is likely to be effective should remain the rule in the circumstances of antibiotic shortage as well as in regular drug distribution.

Previous international data show that dentists account for about 7–10% of the antibiotics prescribed in primary care [13,22]. According to our results, Croatia belongs to the higher end of the antibiotic prescribing scale, taking a 9.6% share in national outpatient antibiotic utilization, although there has been a change in these proportions among some European countries lately. Norway was a good example of rational antibiotic use in dentistry with 8%, but it changed during COVID-19 pandemic, surging to 15% [22]. In Germany, the use of antibiotics in medicine has decreased by 41.6% in a decade, while reduction of dental antibiotic prescriptions was only 13.5%. The percentage of dental prescriptions in relation to all antibiotic prescriptions in Germany has increased from 9.1% in 2012 to 13.6% in 2021 [33], mainly due to a decrease in all antibiotic prescriptions in primary care [34]. These increases have been noticed just recently, and their reasons are yet to be investigated.

Although an international standard for the use of antibiotics in prescriptions per 1000 population has not been empirically established, when applying this common measure for easier comparison to other countries, Croatian dentists prescribed 89 prescriptions/1000 population, a rate that is much higher than in many other investigated countries. For example, dentists in Norway had 3.1 prescriptions/1000 population [35], in Australia the rate of antibiotic use was 33.2 antibiotic per 1000 population, while dentists in the United States had the rate of antibiotic use of 72.6 antibiotic items per 1000 population. The same concerns about high and therefore inappropriate antibiotic prescribing remain when data are expressed in DID [36,37,38]. This result is alarming, and more intense efforts are needed to combat such a high antibiotic rate in dentistry in Croatia. Efforts solely on the graduate educational level are not powerful enough. As seen in the Serbia example, younger dentists are more compliant with the current guidelines, while older dentists are not. Involvement of policymakers is essential, as is shown in the Belgium example [39]. Belgium developed a national plan in collaboration between the nine state major institutions. The plan consisted of 10 strategic objectives and resulted in a significant decrease in antibiotic utilization in a time span of two decades. Sweden also managed to decrease dental antibiotic prescriptions by 31% with governmental support [40]. An Australian study demonstrated that a specifically designed, targeted intervention can reduce inappropriate antibiotic prescribing and improve guideline adherence [41].

The impact of the COVID-19 pandemic on dental prescriptions in Croatia was seen in increased antibiotic utilization in the 2020 pandemic year, and less for 2021. A statistically significant change in antibiotic prescribing practices during the pandemic period was noted for co-amoxiclav, while the highest increase in trend was recorded for azithromycin (39.3%). Norway recorded a significant increase in dental antibiotic prescribing during the pandemic due to defensive prescribing in situations where dental consultations could not be provided under pandemic circumstances. However, the pandemic lockdowns have long-since ended, whereas this trend of increased antibiotic prescription in dentistry continues to persist [42]. In England, the total number of antibiotic items dispensed for the period of April to July was 25% higher in 2020 compared to 2019 [43]. The impact of the pandemic is still evident across various healthcare fields, and ongoing monitoring is recommended to enable timely interventions.

When following mentioned changes in azithromycin prescribing, there were no significant changes found. However, there is no rational explanation for such azithromycin use in dental medicine. Azithromycin is not the first-line antibiotic in dentistry according to various guidelines and recommendations [1,2], and only a few countries use it among the top five antibiotics for odontogenic infections. These include the USA, Brazil, and Belgium [44]. One of the possible explanations for such a non-justified high rate of azithromycin prescribing could be found in recent changes of antibiotic prophylaxis for patients at risk for infective endocarditis, where clindamycin is substituted with azithromycin due to serious adverse reactions. When interpreting this change in guidelines, we noticed among our students and colleagues that the switch from clindamycin to azithromycin was simply mirrored in the guidelines for odontogenic infections, which is a misconduct. Azithromycin is not effective for treating odontogenic infections due to its antibacterial spectrum and low efficacy in bone, and this should be addressed and pointed out.

Significant opportunities exist for the global dental community to contribute to international efforts to tackle antibiotic resistance, including by changing from broad-spectrum antibiotics (e.g., amoxicillin/clavulanate) to narrower-spectrum antibiotics (e.g., phenoxymethylpenicillin) and by reducing the use of WHO ‘Watch’ antibiotics (e.g., azithromycin) [28]. The most recent review proposed four pillars for focused intervention that can be applied in different dental settings to ensure best practices for the successful implementation of rational antimicrobial prescribing in dental medicine: (1) promote education on correct antimicrobial prescriptions at the graduate and post-graduate level, (2) ensure the internalization of responsibility by understanding that each and every one of our antibiotic prescriptions will have a wider clinical and environmental effect, (3) recognize recurring counter-productive practices, and (4) address them through continued education and tailored rational antimicrobial prescription models [42].

Prescribing is a multifaceted process, distinct from clinical dentistry, requiring skills in information gathering, diagnosis, clinical decision-making, precise communication, and ongoing review. There is always a strong recommendation to follow recent guidelines, as for antibiotic prophylaxis of infective endocarditis, for example [45]. Errors and near misses can occur at any stage, potentially causing patient harm. Practitioners must engage in self-reflection to continually improve and minimize errors. Ultimately, prescribing focuses on patient-centered care and shared decision-making, with decisions based on the best evidence to ensure the safe and rational use of medicines for optimal patient outcomes.

### Limitations of the Study

A limitation of our study is the absence of patient data and information on specific indications, such as the type of odontogenic infection, in our database. Consequently, we were unable to assess the overall quality of dental antibiotic prescribing. Additionally, information on private prescriptions is generally absent, and there is no information on whether they were issued by general practitioners or dentists; however, their impact at the national level appears negligible since only 8.4% of all prescriptions are private. Another limitation is the lack of data on OTC (over the counter) medicine consumption. Despite OTC prescriptions accounting for 10.5% of national medication consumption, the current representation without OTC prescriptions still reflects a realistic consumption rate because in Croatia antibiotics are not available without prescription, as their prescribing and dispensing are strictly regulated by law.

## 4. Materials and Methods

This retrospective study focused on analysis of data of antibiotic classes from all Croatian dental medicine doctors (dentists) prescriptions. The data were obtained for the years 2014 to 2023 from the Croatian Health Insurance Fund (CHIF). The great majority of all prescriptions in Croatia (91.6%) are funded by this central government medical insurance agency [10]. Data used for analysis were the number of prescriptions, the cost of medicines in national currency (EUR), and the number of packages prescribed. The data on private practice prescriptions or medicines dispensed to in-hospital patients were not collected. According to the World Health Organization (WHO), a methodology based on the defined daily dose (DDD)/1000 inhabitants/day (DID) and Anatomical Therapeutic Chemical classification (ATC) was used for the quantitative analysis [46]. To calculate a standardized measure of medicine use at the national level, the DID formula was used.

The official national data provider, the Croatian Bureau of Statistics, provided the mid-year population estimates [9]. The Croatian Institute of Public Health (CIPH) provided the number of licensed dentists and the number of patients admitted for dental or oral health reasons for each year included in this study [47].

The collected data were analyzed using the statistical program Statistica, version 14.0.0.15 (TIBCO Software Inc. Santa Clara, CA, USA) and Microsoft^®^ Excel 2016 software was used to analyze annual prescriptions. Descriptive statistics were used to present the results as means and percentages and are presented numerically in tables, or graphically in figures. The paired *t*-test was used for comparisons among time periods to test the differences between specific means (prescribed drugs in time intervals) due to the central limit theorem. Intervals of 2 years were compared (from 2014/2015 to 2022/2023). To present the change in prescribing trend in successive intervals, each interval was compared to the following one. To present the change during the whole observation period, the first interval was compared to the last one.

The data excluded any personal information of the patients or the dentists, ensuring complete personal information protection and anonymity. The Ethical Board of the University of Zagreb School of Dental Medicine reviewed and approved the study under approval No. 05-PA-30-XI-11/2019.

## 5. Conclusions

The proportion of antibiotic use among dentists in Croatia is high and inappropriate. More than half of the prescribed antibiotics (63%) were wide spectrum co-amoxiclav, which is the third option in systemic antibiotic treatment of odontogenic infection. Interruption in the production of the first recommended antibiotic in dentistry has led to increased prescribing of broad-spectrum antibiotics, which additionally reinforced the preexisting upward trends in dental prescribing in Croatia. Antibiotic prescribing must remain evidence-based after careful consideration of non-medicine management options, consideration of the individual’s clinical conditions, risks and benefits, drug and/or disease interactions, as well as monitoring outcomes and minimizing medicine misuse.

To promote the rational use of antibiotics in outpatient dental care, continuously updated, evidence-based guidelines are essential. These should include clear recommendations on: (a) when antibiotics are needed or not needed, (b) which antibiotics should be prescribed, and (c) appropriate dosing and duration of therapy. Ongoing monitoring and evaluation are necessary to reverse current trends through patient and dentist education.

## Figures and Tables

**Table 1:** National medication consumption in dental medicine between 2014 and 2023 and their contribution to the overall national drugs consumption.

Year	Cost (EUR)/Contribution to the National Financial Burden	Number of Overall Issued Prescriptions
2014	2,108,636.07/(0.46%)	425,385
2015	1,893,366.23/(0.41%)	439,737
2016	1,884,353.39/(0.39%)	459,718
2017	1,766,086.82/(0.35%)	456,499
2018	1,672,978.25/(0.31%)	449,683
2019	1,683,357.15/(0.29%)	451,728
2020	1,751,830.89/(0.28%)	467,947
2021	1,792,705.64/(0.25%)	479,427
2022	1,704,723.32/(0.23%)	466,250
2023	1,737,444.36/(data not available)	451,513

**Table 2:** The prescribed antibiotic between 2014 and 2023 by the annual number of prescriptions (percentage of total antibiotic prescriptions).

ATC Generic Name	Number of Antibiotic Prescriptions (Percentage)									
	2014	2015	2016	2017	2018	2019	2020	2021	2022	2023
J01CR02 amoxicillin + clavulanic acid	186,698 (53.92%)	194,123 (55.12%)	205,411 (56.42%)	209,632 (57.67%)	212,860 (58.77%)	213,156 (59.67%)	221,922 (61.27%)	229,072 (61.84%)	204,971 (57.43%)	217,162 (63.17%)
J01FF01 clindamycin	44,805 (12.94%)	45,391 (12.89%)	46,362 (12.73%)	44,544 (12.25%)	43,696 (12.06%)	43,049 (12.05%)	44,333 (12.24%)	43,602 (11.77%)	48,498 (13.59%)	46,005 (13.38%)
J01CA04 amoxicillin	52,492 (15.16%)	50,863 (14.44%)	50,349 (13.83%)	48,077 (13.23%)	45,714 (12.62%)	43,184 (12.09%)	39,796 (10.99%)	40,260 (10.87%)	47,004 (13.17%)	33,544 (9.76%)
P01AB01 metronidazole	33,330 (9.63%)	35,049 (9.95%)	36,923 (10.14%)	37,254 (10.25%)	38,284 (10.57%)	38,391 (10.75%)	35,870 (9.9%)	37,308 (10.07%)	35,870 (10.05%)	28,142 (8.19%)
J01FA10 azitromycine	5311 (1.53%)	5473 (1.55%)	5398 (1.48%)	5767 (1.59%)	5794 (1.6%)	5872 (1.64%)	8181 (2.26%)	8892 (2.4%)	8992 (2.52%)	7887 (2.29%)
J01DB01 cephalexin	18,092 (5.23%)	16,516 (4.69%)	15,220 (4.18%)	13,796 (3.8%)	11,475 (3.17%)	9642 (2.7%)	8631 (2.38%)	8056 (2.17%)	8287 (2.32%)	7519 (2.19%)
J01DC02 cefuroxime	917 (0.26%)	996 (0.28%)	1018 (0.28%)	977 (0.27%)	1045 (0.29%)	987 (0.28%)	795 (0.22%)	744 (0.2%)	674 (0.19%)	742 (0.22%)
J01DD08 cefixime	69 (0.02%)	62 (0.02%)	56 (0.02%)	160 (0.04%)	244 (0.07%)	250 (0.07%)	280 (0.08%)	275 (0.07%)	411 (0.12%)	682 (0.2%)
J01FA09 clarithromycin	541 (0.15%)	556 (0.15%)	426 (0.11%)	563 (0.15%)	579 (0.15%)	519 (0.14%)	533 (0.14%)	506 (0.13%)	671 (0.18%)	628 (0.18%)
J01FA01 erithromycin	1153 (0.33%)	1146 (0.33%)	1093 (0.3%)	969 (0.27%)	900 (0.25%)	777 (0.22%)	682 (0.19%)	709 (0.19%)	576 (0.16%)	529 (0.15%)
J01AA02 doxycycline	642 (0.19%)	595 (0.17%)	531 (0.15%)	574 (0.16%)	554 (0.15%)	513 (0.14%)	492 (0.14%)	383 (0.1%)	383 (0.11%)	295 (0.09%)
J01CE02 phenoxymethylpenicillin	217 (0.06%)	747 (0.21%)	816 (0.22%)	733 (0.2%)	570 (0.16%)	460 (0.13%)	313 (0.09%)	317 (0.09%)	219 (0.06%)	238 (0.07%)
J01DD13 cefpodoxime	1 (0.01%)	5 (0.01%)	3 (0.01%)	4 (0.01%)	19 (0.01%)	27 (0.01%)	38 (0.01%)	57 (0.02%)	85 (0.02%)	123 (0.04%)
J01MA02 ciprofloxacin	97 (0.03%)	84 (0.02%)	88 (0.02%)	88 (0.02%)	83 (0.02%)	69 (0.02%)	56 (0.02%)	58 (0.02%)	50 (0.01%)	81 (0.02%)
J01MA06 norfloxacin	87 (0.03%)	61 (0.02%)	70 (0.02%)	69 (0.02%)	94 (0.03%)	81 (0.02%)	84 (0.02%)	64 (0.02%)	70 (0.02%)	54 (0.02%)
J01EE01 sulfamethoxazole + trimethoprim	386 (0.11%)	357 (0.1%)	259 (0.07%)	230 (0.06%)	188 (0.05%)	169 (0.04%)	134 (0.03%)	61 (0.01%)	75 (0.02%)	45 (0.01%)
J01XE01 nitrofurantoin	23 (0.01%)	40 (0.02%)	22 (0.01%)	27 (0.01%)	36 (0.01%)	44 (0.02%)	47 (0.02%)	52 (0.02%)	39 (0.02%)	33 (0.01%)
J01XX01 fosfomycin	0 (0%)	0 (0%)	0 (0%)	6 (0.01%)	16 (0.01%)	11 (0.01%)	18 (0.01%)	19 (0.01%)	21 (0.01%)	27 (0.01%)
J01MA12 levofloxacin	6 (0.01%)	8 (0.01%)	3 (0.01%)	3 (0.01%)	10 (0.01%)	11 (0.01%)	6 (0.01%)	5 (0.01%)	7 (0.01%)	9 (0.01%)
J01MA14 moxifloxacin	12 (0.01%)	6 (0.01%)	2 (0.01%)	2 (0.01%)	7 (0.01%)	0 (0%)	7 (0.01%)	2 (0.01%)	2 (0.01%)	5 (0.01%)
J01CE10 benzathine phenoxymethylpenicillin	1341 (0.39%)	85 (0.02%)	52 (0.01%)	12 (0.01%)	26 (0.01%)	29 (0.01%)	13 (0.01%)	3 (0.01%)	0 (0%)	0 (0%)
J01DD14 ceftibuten	5 (0.01%)	6 (0.01%)	2 (0.01%)	0 (0%)	0 (0%)	0 (0%)	0 (0%)	0 (0%)	0 (0%)	0 (0%)
Total	346,225 (100%)	352,169 (100%)	364,104 (100%)	363,487 (100%)	362,194 (100%)	357,241 (100%)	362,231 (100%)	370,445 (100%)	356,905 (100%)	343,750 (100%)

**Table 3:** The prescribed antibiotics in DID between 2014 and 2023.

ATC Generic Name	DID 2014	DID 2015	DID 2016	DID 2017	DID 2018	DID 2019	DID 2020	DID 2021	DID 2022	DID 2023
J01AA02 doxycycline	0.013	0.012	0.011	0.011	0.011	0.01	0.009	0.007	0.007	0.005
J01CA04 amoxicillin	0.19	0.19	0.19	0.18	0.174	0.16	0.15	0.16	0.185	0.14
J01CE02 phenoxymethylpenicillin	0.0025	0.0013	0.0014	0.0012	0.001	0.0008	0.0005	0.0005	0.0004	0.0004
J01CR02 amoxicillin + clavulanic acid	1.15	1.22	1.28	1.3	1.36	1.38	1.47	1.54	1.39	1.47
J01DB01 cephalexin	0.05	0.05	0.05	0.04	0.04	0.031	0.029	0.028	0.029	0.026
J01DC02 cefuroxime	0.0056	0.006	0.0062	0.0063	0.0065	0.0062	0.005	0.0048	0.0043	0.0049
J01DD08 cefixime	0.0002	0.0002	0.0002	0.0006	0.0009	0.0009	0.001	0.001	0.0016	0.0025
J01DD13 cefpodoxime	0	0.00002	0.00001	0.00002	0.00004	0.00008	0.0001	0.00017	0.00028	0.0004
J01EE01 sulfamethoxazole + trimethoprim	0.0058	0.0054	0.0039	0.0035	0.0026	0.0025	0.002	0.0009	0.001	0.0008
J01FA01 erithromycin	0.0038	0.0039	0.0037	0.0035	0.0032	0.0028	0.0027	0.0027	0.0021	0.0021
J01FA09 clarithromycin	0.0044	0.0046	0.0034	0.0046	0.0048	0.0043	0.0044	0.0044	0.0057	0.0053
J01FA10 azitromycin	0.01	0.011	0.011	0.012	0.012	0.013	0.016	0.02	0.021	0.018
J01FF01 clindamycin	0.13	0.13	0.13	0.13	0.13	0.128	0.138	0.136	0.154	0.145
J01MA02 ciprofloxacin	0.0004	0.0004	0.00039	0.00039	0.00035	0.0003	0.00025	0.00026	0.00023	0.0004
J01MA06 norfloxacin	0.00066	0.00043	0.00055	0.00059	0.0008	0.0007	0.00068	0.00056	0.00062	0.00044
J01MA12 levofloxacin	0.000058	0.000026	0.000028	0.00002	0.000087	0.0001	0.000047	0.000056	0.000056	0.000064
J01MA14 moxifloxacin	0.000067	0.000027	0.000009	0.000009	0.00006	0	0.000056	0.000019	0.000014	0.000025
P01AB01 metronidazole	0.07	0.08	0.08	0.08	0.08	0.082	0.085	0.081	0.079	0.062

## Data Availability

The original contributions presented in the study are included in the article, further inquiries can be directed to the corresponding author.

## References

[1] Teughels W., Feres M., Oud V., Martín C., Matesanz P., Herrera D. (2020). Adjunctive Effect of Systemic Antimicrobials in Periodontitis Therapy: A Systematic Review and Meta-Analysis. J. Clin. Periodontol..

[2] Segura-Egea J.J., Gould K., Şen B.H., Jonasson P., Cotti E., Mazzoni A., Sunay H., Tjäderhane L., Dummer P.M.H. (2018). European Society of Endodontology Position Statement: The Use of Antibiotics in Endodontics. Int. Endod. J..

[3] Thornhill M.H., Dayer M., Prendergast B.D., Lockhart P., Baddour L. (2023). Antibiotic Prophylaxis in Dentistry. Clin. Infect. Dis..

[4] Romandini M., De Tullio I., Congedi F., Kalemaj Z., D’Ambrosio M., Laforí A., Quaranta C., Buti J., Perfetti G. (2019). Antibiotic Prophylaxis at Dental Implant Placement: Which Is the Best Protocol? A Systematic Review and Network Meta-Analysis. J. Clin. Periodontol..

[5] Šutej I., Lepur D., Božić D., Pernarić K. (2021). Medication Prescribing Practices in Croatian Dental Offices and Their Contribution to National Consumption. Int. Dent. J..

[6] Roganović J., Barać M. (2024). Rational Antibiotic Prescribing Is Underpinned by Dental Ethics Principles: Survey on Postgraduate and Undergraduate Dental Students’ Perceptions. Antibiotics.

[7] Săndulescu O., Săndulescu M. (2023). The 5Ds of Optimized Antimicrobial Prescription in Dental Medicine. Germs.

[8] European Commission Communication from the Commission to the European Parliament, the Council, the European Economic and Social Committee and the Committee of the Regions: Addressing Medicine Shortages in the EU 2023. https://eur-lex.europa.eu/legal-content/EN/TXT/?uri=COM%3A2023%3A672%3AREV1.

[9] Croatian Bureau of Statistics Population Estimate. https://podaci.dzs.hr/en/statistics/population/population-estimate/.

[10] Draganić P., Oštarčević S., Škribulja M. (2022). Potrošnja Lijekova u Hrvatskoj 2017–2021.

[11] Šutej I., Lepur D., Bašić K., Šimunović L., Peroš K. (2023). Changes in Medication Prescribing Due to COVID-19 in Dental Practice in Croatia-National Study. Antibiotics.

[12] Šimundić Munitić M., Šutej I., Ćaćić N., Tadin A., Balić M., Bago I., Poklepović Peričić T. (2021). Knowledge and Attitudes of Croatian Dentists Regarding Antibiotic Prescription in Endodontics: A Cross-Sectional Questionnaire-Based Study. Acta Stomatol. Croat..

[13] Perić M., Perković I., Romić M., Simeon P., Matijević J., Mehičić G.P., Krmek S.J. (2015). The Pattern of Antibiotic Prescribing by Dental Practitioners in Zagreb, Croatia. Cent. Eur. J. Public Health.

[14] Bjelovucic R., Par M., Rubcic D., Marovic D., Prskalo K., Tarle Z. (2019). Antibiotic Prescription in Emergency Dental Service in Zagreb, Croatia—A Retrospective Cohort Study. Int. Dent. J..

[15] Sović J., Šegović S., Tomasić I., Pavelić B., Šutej I., Anić I. (2020). Antibiotic Administration Along with Endodontic Therapy in the Republic of Croatia: A Pilot Study. Acta Stomatol. Croat..

[16] Segura-Egea J.J., Velasco-Ortega E., Torres-Lagares D., Velasco-Ponferrada M.C., Monsalve-Guil L., Llamas-Carreras J.M. (2010). Pattern of Antibiotic Prescription in the Management of Endodontic Infections amongst Spanish Oral Surgeons. Int. Endod. J..

[17] Deniz-Sungur D., Aksel H., Karaismailoglu E., Sayin T.C. (2020). The Prescribing of Antibiotics for Endodontic Infections by Dentists in Turkey: A Comprehensive Survey. Int. Endod. J..

[18] Tuculina M.J., Diaconu O.A., Madalina C., Gheorghiță L.M., Bătăiosu M., Dragomir L., Nicola A.G., Cumpătă C.N., Dăguci C., Turcu A. (2022). An Observational Study: Use of Systemic Antibiotics for Endodontic Infections Treatment. J. Dent. Health Oral Res..

[19] Domínguez-Domínguez L., López-Marrufo-Medina A., Cabanillas-Balsera D., Jiménez-Sánchez M.C., Areal-Quecuty V., López-López J., Segura-Egea J.J., Martin-González J. (2021). Antibiotics Prescription by Spanish General Practitioners in Primary Dental Care. Antibiotics.

[20] Drobac M., Otasevic K., Ramic B., Cvjeticanin M., Stojanac I., Petrovic L. (2021). Antibiotic Prescribing Practices in Endodontic Infections: A Survey of Dentists in Serbia. Antibiotics.

[21] Khalil D., Baranto G., Lund B., Hultin M. (2022). Antibiotic Utilization in Emergency Dental Care in Stockholm 2016: A Cross Sectional Study. Acta Odontol. Scand..

[22] Tousi F., Al Haroni M., Lie S.A., Lund B. (2023). Antibiotic Prescriptions among Dentists across Norway and the Impact of COVID-19 Pandemic. BMC Oral Health.

[23] Baudet A., Kichenbrand C., Pulcini C., Descroix V., Lesclous P., Thilly N., Clément C., Guillet J. (2020). Antibiotic Use and Resistance: A Nationwide Questionnaire Survey among French Dentists. Eur. J. Clin. Microbiol. Infect. Dis..

[24] Mainjot A., D’Hoore W., Vanheusden A., Van Nieuwenhuysen J.-P. (2009). Antibiotic Prescribing in Dental Practice in Belgium. Int. Endod. J..

[25] Tolksdorf K., Freytag A., Bleidorn J., Markwart R. (2022). Antibiotic Use by Dentists in Germany: A Review of Prescriptions, Pathogens, Antimicrobial Resistance and Antibiotic Stewardship Strategies. Community Dent. Health.

[26] Teoh L., Hopcraft M., McCullough M., Manski-Nankervis J.-A., Biezen R. (2023). Exploring the Appropriateness of Antibiotic Prescribing for Dental Presentations in Australian General Practice—A Pilot Study. Br. J. Clin. Pharmacol..

[27] Ramanathan S., Yan C.H., Hubbard C., Calip G.S., Sharp L.K., Evans C.T., Rowan S., McGregor J.C., Gross A.E., Hershow R.C. (2023). Changes in Antibiotic Prescribing by Dentists in the United States, 2012–2019. Infect. Control Hosp. Epidemiol..

[28] Thompson W., Teoh L., Hubbard C.C., Marra F., Patrick D.M., Mamun A., Campbell A., Suda K.J. (2022). Patterns of Dental Antibiotic Prescribing in 2017: Australia, England, United States, and British Columbia (Canada). Infect. Control Hosp. Epidemiol..

[29] Cohen R., Pettoello-Mantovani M., Giardino I., Carrasco-Sanz A., Somekh E., Levy C. (2023). The Shortage of Amoxicillin: An Escalating Public Health Crisis in Pediatrics Faced by Several Western Countries. J. Pediatr..

[30] Poole N.M., Lee B.R., Kronman M.P., Smith M.J., Patel S.J., Olivero R., Wattles B.A., Herigon J., Wirtz A., El Feghaly R.E. (2024). Ambulatory Amoxicillin Use for Common Acute Respiratory Infections during a National Shortage: Results from the SHARPS-OP Benchmarking Collaborative. Am. J. Infect. Control.

[31] Khazanchi R., Brewster R., Butler A., O’Meara D., Bagchi D.P., Michelson K. (2023). 1652. Impact of the 2022–2023 Amoxicillin Shortage on Antibiotic Prescribing for Acute Otitis Media: A Regression Discontinuity Study. Open Forum Infect. Dis..

[32] European Commission EU Steps up Action to Prevent Shortages of Antibiotics. https://ec.europa.eu/commission/presscorner/detail/en/ip_23_3890.

[33] Albrecht H., Schiegnitz E., Halling F. (2024). Facts and Trends in Dental Antibiotic and Analgesic Prescriptions in Germany, 2012–2021. Clin. Oral Investig..

[34] Gradl G., Kieble M., Nagaba J., Schulz M. (2022). Assessment of the Prescriptions of Systemic Antibiotics in Primary Dental Care in Germany from 2017 to 2021: A Longitudinal Drug Utilization Study. Antibiotics.

[35] Kjome R.L.S., Bjønnes J.A.J., Lygre H. (2021). Changes in Dentists’ Prescribing Patterns in Norway 2005–2015. Int. Dent. J..

[36] Struyf T., Vandael E., Leroy R., Mertens K., Catry B. (2019). Antimicrobial Prescribing by Belgian Dentists in Ambulatory Care, from 2010 to 2016. Int. Dent. J..

[37] Smith A., Al-Mahdi R., Malcolm W., Palmer N., Dahlen G., Al-Haroni M. (2020). Comparison of Antimicrobial Prescribing for Dental and Oral Infections in England and Scotland with Norway and Sweden and Their Relative Contribution to National Consumption 2010–2016. BMC Oral Health.

[38] Teoh L., Stewart K., Marino R., McCullough M. (2018). Current Prescribing Trends of Antibiotics by Dentists in Australia from 2013 to 2016. Part 1. Dent. J..

[39] Bruyndonckx R., Coenen S., Hens N., Vandael E., Catry B., Goossens H. (2021). Antibiotic Use and Resistance in Belgium: The Impact of Two Decades of Multi-Faceted Campaigning. Acta Clin. Belg..

[40] Lund B., Cederlund A., Hultin M., Lundgren F. (2020). Effect of Governmental Strategies on Antibiotic Prescription in Dentistry. Acta Odontol. Scand..

[41] Teoh L., Stewart K., Marino R.J., McCullough M.J. (2021). Improvement of Dental Prescribing Practices Using Education and a Prescribing Tool: A Pilot Intervention Study. Br. J. Clin. Pharmacol..

[42] Săndulescu O., Preoțescu L.L., Streinu-Cercel A., Şahin G.Ö., Săndulescu M. (2024). Antibiotic Prescribing in Dental Medicine-Best Practices for Successful Implementation. Trop. Med. Infect. Dis..

[43] Shah S., Wordley V., Thompson W. (2020). How Did COVID-19 Impact on Dental Antibiotic Prescribing across England?. Br. Dent. J..

[44] Odeh N.-D., Babkair H., Abu-Hammad S., Borzangy S., Abu-Hammad A., Abu-Hammad O. (2020). COVID-19: Present and Future Challenges for Dental Practice. Int. J. Environ. Res. Public Health.

[45] ESC Scientific Document Group (2023). 2023 ESC Guidelines for the management of endocarditis. Eur. Heart J..

[46] World Health Organization Collaborating Centre for Drug Statistics Methodology ATC/DDD Index. https://atcddd.fhi.no/atc_ddd_index/.

[47] Hrvatski Zavod za Javno Zdravstvo Hrvatski Zdravstveno-Statistički Ljetopis za 2020—Tablični Podaci. https://www.hzjz.hr/hrvatski-zdravstveno-statisticki-ljetopis/hrvatski-zdravstveno-statisticki-ljetopis-za-2020-tablicni-podaci/.

